# Reversal of angiotensin ll-induced β-cell dedifferentiation via inhibition of NF-κb signaling

**DOI:** 10.1186/s10020-018-0044-3

**Published:** 2018-08-14

**Authors:** Hong Chen, Wenjun Zhou, Yuting Ruan, Lei Yang, Ningning Xu, Rongping Chen, Rui Yang, Jia Sun, Zhen Zhang

**Affiliations:** 1 Department of Endocrinology, Zhujiang Hospital, Southern Medical University, 253, Gongyedadao Middle, Guangzhou, Guangdong 510282 People’s Republic of China; 2 Department of Nephrology, Zhujiang Hospital, Southern Medical University, 253, Gongyedadao Middle, Guangzhou, Guangdong 510282 People’s Republic of China

**Keywords:** β-Cell dedifferentiation, Renin-angiotensin system, Angiotensin ll, Type 2 diabetes, NF-κb

## Abstract

**Background:**

Type 2 diabetes mellitus (T2DM) is characterized by pancreatic β-cell failure, which arises from metabolic stress and results in β cell dedifferentiation, leading to β-cell death. Pathological activation of the renin–angiotensin system (RAS) contributes to increase cell stress, while RAS intervention reduces the onset of T2DM in high-risk populations and promotes insulin secretion in rodents. In this study, we investigated whether and how RAS induces β-cell dedifferentiation and the mechanism underlying this process.

**Methods:**

In vitro, with the methods of quantitative real-time reverse transcriptase-PCR (qRT-PCR) and western blotting, we examined the change of cell identity-related gene expression, progenitor like gene expression, cellular function, and nuclear factor kappa b (NF-κb) signaling activity in β cell lines after exposure to angiotensin II (AngII) and disruption of RAS. In vivo, parallel studies were performed using db/db mice. Related protein expression was detected by Immunofluorescence analysis.

**Result:**

Activation of RAS induced dedifferentiation and impaired insulin secretion, eventually leading to β-cell failure. Mechanistically, Angll induced β-cell dedifferentiation via NF-κb signaling, while treatment with lrbesartan and sc-514 reversed the progenitor state of β cells.

**Conclusion:**

The present study found that RAS might induce β-cell dedifferentiation via angiotensin II receptor type 1 activation, which was promoted by NF-κb signaling. Therefore, blocking RAS or NF-kb signaling efficiently reversed the dedifferentiated status of β cells, suggesting a potential therapy for patients with type 2 diabetes.

**Electronic supplementary material:**

The online version of this article (10.1186/s10020-018-0044-3) contains supplementary material, which is available to authorized users.

## Background

Pancreatic β-cell failure underlies the progressive development of type 2 diabetes, accompanied by the functional decline of β cells which commonly arises from metabolic stress and dedifferentiation (Swisa et al., 2017; Weir et al., 2013). Increasing evidence shows that the pathological stimulation of the renin–angiotensin system (RAS) is associated with type 2 diabetes and that RAS inhibitors, either Angiotensin (Ang) II type 1 receptor blockers (ARBs) or Angll-converting enzyme inhibitors (ACEi), delay new onset-type 2 diabetes in high-risk populations (Abuissa et al., 2005; Prisant 2004). The local components of RAS have been detected in many tissues and organs, including the pancreas, adipose tissue, skeletal muscle, and brain, suggesting an important physiological role of systemic RAS (Chan et al., 2017; Grobe and Rahmouni 2012; Jones and Woods 2003; Mateos et al., 2011). Angll, a powerful RAS component, not only diminishes islet blood flow, but also is involved in increased oxidative stress, proinflammatory cytokines, and islet fibrosis (Ihoriya et al., 2014; Xu et al., 2011). Furthermore, studies show that Angll contributes to impaired β-cell function in Angll-infused mice, while the detrimental effect is independent of vasoconstriction, implying a complex mechanism underlying Angll-induced β-cell dysfunction (Sauter et al., 2015). Mechanistically, inhibition of RAS promotes insulin secretion and β-cell function in diabetic mice (Huang et al., 2007; Saitoh et al., 2009). However, although previous studies have shed some light on the relationship between RAS and type 2 diabetes, the underlying role of RAS remains incompletely understood.

Emerging evidence shows that β-cell failure can develop through different mechanisms, including oxidative stress, endoplasmic reticulum stress, hypoxic stress, and the induction of proinflammatory cytokines (Sauter et al., 2015; Chan et al., 2017; Cnop et al., 2017). In response to the stressors, β cells become dedifferentiated, reverting to a progenitor-like stage or converting into other types of pancreatic cells, such as α, δ, and pancreatic polypeptide cells (Pp cells) (Talchai et al., 2012a). Hence, it has been recognized recently that loss of the fully differentiated state is a potential mechanism underlying compromised β-cell function in type 2 diabetes (Spijker et al., 2015; Steven et al., 2016).

In *Foxo1* knockout mice, metabolic stress triggers the loss of β cells-related transcription factors PDX1 and MAFA, and increases the expression of endocrine progenitor markers NGN3 and OCT4. These changes lead to the loss of mature β cell identity (Talchai et al., 2012a). Consistently, multiple studies have found β-cell dedifferentiation and increased progenitor cell marker expression in patients with type 2 diabetes (Cinti et al., 2016; Guo et al., 2013b). Conversely, RAS blockers preserve β-cell function and attenuate oxidative stress and NADPH oxidase activation in pancreatic β cells (Saitoh et al., 2009).

Recently, clinical trial and experimental evidence have shown that nuclear factor kappa b (NF-κb)-mediated inflammation is associated with the cardioprotective effects of RAS inhibitors (Huynh et al., 2014; Thomas et al., 2013), and AngII has been reported to stimulate NF-κb activity in different types of cells (Pandey et al., 2015; Xu et al., 2011). NF-κB was reported to be a major transcription factor that positively regulates angiotensin II type 1 receptor (*AT1R*) gene expression (Daniluk et al., 2012; Wolf et al., 2002). The inactivated NF-κB complex is localized in the cytoplasm and includes the DNA binding p65 subunits and an inhibitory subunit, IкBα, which is bound to p65. ln response to stimulation, phosphorylation of the IкB kinase (IKK) complex releases IkBα from the complex and promotes the nuclear translocation of p65, which regulates gene transcription (Gilmore 2006). Moreover, inhibition of IкB-kinase prevents Angll-induced upregulation of proinflammatory cytokines (interleukin (IL)-1b and IL-6) in human islets (Sauter et al., 2015). Taken together, this evidence suggests an interaction between RAS and NF-κB in the development of diabetes.

In summary, these findings allowed us to formulate a hypothesis that RAS activation plays an important role in β cell dedifferentiation, while interference with RAS or NF-κb signaling could be an efficient way to reverse β-cell dedifferentiation.

## Methods

### Pancreatic β-cell line culture

The pancreatic β-cell line Min6 (Mouse insulinoma 6) (Miyazaki et al., 1990) and INS-1 (rat insulinoma cells) (Zhang et al., 2014a) were cultured in Roswell Park Memorial Institute (RPMI) 1640 (Invitrogen) medium containing 11.1 mmol/L glucose, supplemented with 10% fetal bovine serum (FBS), 10 mmol/L HEPES, 2 mmol/L L-Glutamine, 50 U/mL penicillin, 50 mg/mL streptomycin and 50 mmol/L 2-mercaptoethanol at 37 °C in a humidified atmosphere of 95% air and 5% CO_2_. We conducted experiments when the cells reach 70% confluence. For the experiments, we subcultured the cells in a 6-well plate when the cells reached 70% confluence. Cells were cultured in 1640 medium containing 11.1, 22.2, or 33.3 mM glucose and were treated with or without 10 μmol/L irbesartan for 24 h. For Angll-treatment experiments, pancreatic β cells were cultured with or without 1 μmol/L Angll in the presence or absence of 20 μmol/L sc-514, an NF-κb signaling inhibitor, or 10 μmol/L Irbesartan for 48 h. The effect of Ang II on NF-κb signaling in β cells was determined at 0.1, 1, and 10 mol/L of Ang II.

### Glucose-stimulated insulin secretion (GSIS)

Glucose-starved cell preparation was conducted in Krebs–Ringer bicarbonate HEPES (KRBH) buffer (115 mM NaCl, 5 mM KCl, 1 mM MgCl_2_, 24 mM NaHCO_3_, 2.5 mM CaCl_2_, 10 mM HEPES supplemented with 0.5% bovine serum albumin (BSA)) for 45 min. The cells were incubated in basal glucose conditioned KRBH buffer containing 3 mM glucose for 1 h and then incubated in glucose stimulation KRBH buffer containing 25 mM glucose for 1 h (Tuo et al., 2011). Before treatment with different media, the cells were washed twice with phosphate-buffered saline (PBS), and the media were collected for ultra-sensitive enzyme-linked immunosorbent assay (ELISA) analysis (Mercodia, Sweden).

### Animals and treatments

Eight-week-old male C57BL/KsJ-db/db mice and wild-type littermate controls (C57BL/KsJ-db/m mice) were purchased from the Model Animal Research Center of Nanjing University. All the animal protocols were approved by the Ethics Committee for the use of Experimental Animals at Southern Medical University. All mice were maintained in a specific pathogen-free environment and housed in clean cages in groups of two animals per cage, with appropriate temperature and humidity and 12 h/12 h light/dark cycles. After 1 week of acclimatization, non-fasting blood glucose levels in 9-week-old db/db mice were > 16.7 mmol/L, with symptoms of polyuria, polydipsia, and polyphagia. The mice at 9 weeks of age were randomly divided into five groups: (1) Normal group [db/m mice injected with PBS subcutaneously; *n* = 8], (2) vehicle control [db/db mice were injected with PBS subcutaneously; *n* = 8], mice in remaining three groups were injected with human AngII (60 μg/kg; A9525; Sigma) subcutaneously twice a day for 4 weeks, (3) AngII [Angll injection; *n* = 8], (4) AngII+IRB [Angll injection and Irbesartan (IRB; 50 mg/kg; S1507; Sellect) was administered through oral gavage for 4 weeks; *n* = 8], (5) AngII+sc-514 [Angll injection and sc-514 (IRB; 30 mg/kg; S4907; Sellect) was administered through oral gavage for 4 weeks; *n* = 8]. The body weight and blood glucose (the sample for which was withdrawn from the tail vein) were measured and recorded two times weekly during the experiment.

### Glucose tolerance test and insulin measurement

For intraperitoneal glucose tolerance tests (2.5 g glucose/kg body weight), mice were fasted for 5 h, in the indicated groups, before obtaining the blood samples at various time points (0–120 min) from orbital sinus; the blood glucose concentration was determined using a glucometer (Roche, Indianapolis, IN). For insulin measurements, blood samples were centrifuged (2000 rpm for 2 min at 4 °C) and the plasma insulin concentrations were measured using a mouse insulin ELISA kit (Mercodia, Sweden), according to the manufacturer’s instructions.

### Quantitative real-time reverse transcription PCR

Total RNA was extracted from β cells using the TRIzol reagent (Takara Biotechnology, Japan), after which the RNA quantity and purity were evaluated using an NanoDrop 2000 apparatus (Thermo Scientific) and then reverse-transcribed into cDNA using a Reverse Transcription Kit (Takara). For quantitative PCR, the real-time PCR system 7500 (Applied Biosystems) and primers were used to detect gene expression. Gene expression was determined by relative gene expression using the 2^-ΔΔCt^ method.

### Western blotting analysis

Western blotting and immunohistochemistry. Sodium dodecyl sulfate polyacrylamide gel electrophoresis and western blotting were performed as previously described (Zhang et al., 2014a, b) using primary antibodies against Insulin, NGN3, FOXO1, PDX-1, OCT4, phospho-IκBα (p-IκBα), IκBα, phospho-p65 (p-p65), p65, β-actin, and the horseradish peroxidase-labeled secondary antibodies were goat anti-rabbit IgG and goat anti-mouse IgG (Table 1).

**Table 1:** Antibodies used in this study

Primary Antibody; Monoclonal or Polyclonal	Manufacturer	Reactivity	Dilution	Identifier
Rabbit anti-Neurog3; polyclonal	LifeSpan BioSciences	H, M, R	1:200 1:50	LS-C97692; RRID: AB_2282494
Rabbit anti-glucagon; polyclonal	Cell Signaling	H, M, R	1:400	Cat# 2760S RRID:AB_10698611
Mouse anti-insulin; monoclonal	Cell Signaling	H, M, R	1:200	Cat# 8138S, RRID:AB_10949314
Rabbit anti-Pdx; polyclonal	Cell Signaling	H, M, R	1:500 1:200	Cat# 5679, RRID:AB_10706174
Rabbit anti-FoxO1; polyclonal	Cell Signaling	H, M, R	1:500 1:200	Cat# 2880, RRID:AB_2106495
Mouse anti-p-NF-κBp65; Monoclonal	Cell Signaling	H, M, R	1:500	Cat# 3033 RRID:AB_331284
Mouse anti- NF-κBp65; Monoclonal	Cell Signaling	H, M, R	1:500	Cat# 6956, RRID:AB_10828935
Rabbit anti-Oct-4A polyclonal	Cell Signaling	H,M	1:500	Cat# 2890, AB_10841298
Mouse anti- IκBα; monoclonal	Cell Signaling	H, M, R	1:500	Cat# 4814, RRID:AB_390781
Rabbit anti-Phospho-IκBα polyclonal	Cell Signaling	H,M,R	1:250	Cat# 2859, RRID:AB_561111
β-actin	Cell Signaling	H,M	1:1000	Cat# 12262, RRID:AB_2566811

*H* human, *M* mouse, *R* rat

### Immunofluorescence analysis

The pancreatic samples were obtained without saline perfusion (Cheng et al., 2017) and processed for paraffin embedding. Pancreatic sections (5 mm) were dewaxed in dimethylbenzene and rehydrated through graded ethanol series (100, 95, 80, and 70%). Heat-mediated antigen retrieval with citrate buffer was performed and sections were blocked in a 2% BSA solution for 30 min at room temperature. The following primary antibodies were used: anti-insulin, anti-glucagon anti-PDX1, anti-FOXO1 (the primary antibodies were purchased from Cell Signaling), and anti-NGN3 (LifeSpan Biosciences). Sections were incubated with primary antibodies overnight at 4 °C. After washing with PBS, sections were incubated for 40 min at room temperature with secondary antibodies: Alexa Fluor 594 donkey anti-mouse immunoglobulin IgG and Alexa Fluor 488 donkey anti-rabbit IgG (Proteintech). The double staining was captured using a Nikon Y-TV55 fluorescent microscope. Numbers of cells or areas of interest were measured from 3 to 5 mice per group, or 4–5 pancreas sections per mouse for 20 islets. We then measured the positive stained area divided by total islet area (to calculate the staining index) using Image-Pro analyzer software (version 6.0, Media Cybernetics, USA).

### Statistical analysis

Data are expressed as means ± standard error. Statistical analyses were performed using Prism7.0 (GraphPad). For statistical significance of different experimental groups, we used one-way, or repeated measures, analysis of variance (ANOVA). *P* < 0.05 indicated a statistically significant difference.

## Results

### RAS inhibition reverses glucotoxicity-induced compromised β-cell identity

**Fig. 1:**
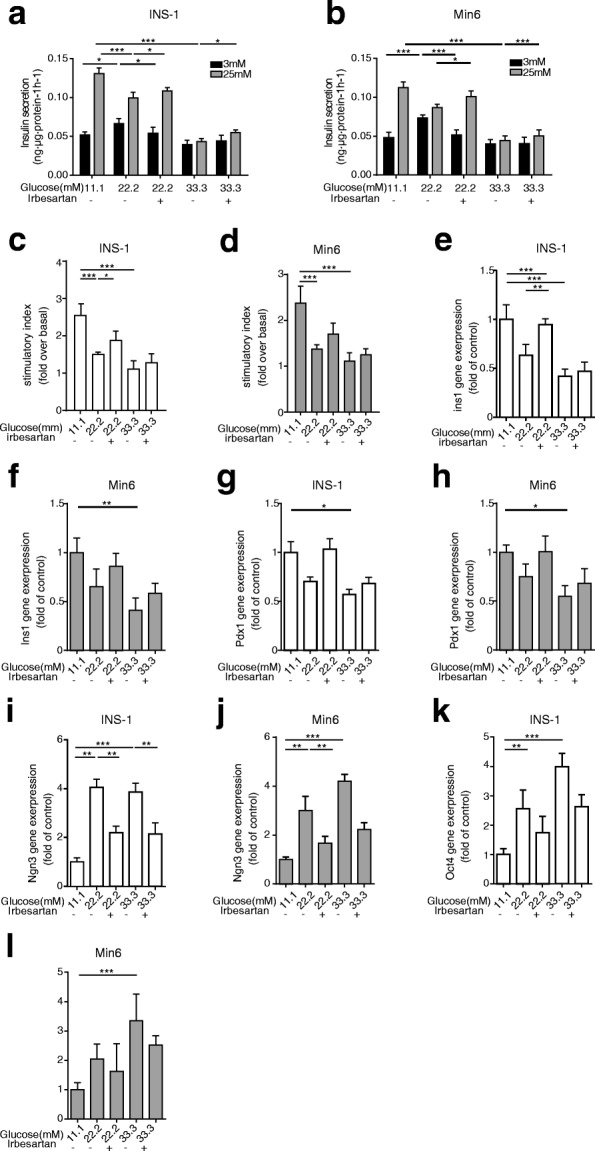
RAS inhibition reverses glucotoxicity-induced compromised β-cell identity. Pancreatic β cell lines were cultured with increasing concentrations of glucose in the presence or absence of irbesartan (IRB, 10 μmol/L) for 24 h. Performing (**a, b**) a GSIS assay to determine (**c, d**) the stimulatory index in Min6 cells and INS-1 cells. qRT-PCR analyses for markers of (**e-h**) β cell identity genes (*Ins1*, *Pdx1*), and (**i-l**) progenitor like cells markers (*Ngn3*, *Oct4*) in β cells. Data are presented as the mean ± SEM of three independent experiments (*n* = 6). *p < 0.05, **p < 0.01, ***p < 0.001, one-way ANOVA

To investigate the effect of RAS blockade on glucose-induced β-cell dysfunction, mouse pancreatic β-cell lines were exposed to increased concentrations of glucose in the presence or absence of Irbesartan (10 μmol/L) for 24 h (Additional file 1: Table S1). β-cell dysfunction was closely related to glucose levels, and exposure to a high glucose environment resulted in a sharp decrease in glucose-stimulated insulin secretion and the stimulatory index in β cells (Fig. 1a-d). By contrast, we observed a slight increase in basal secretion of β cells under 22.2 mmol/L glucose conditions. Furthermore, impaired GSIS was not only associated with decreased mRNA expression of *Pdx1* and *Ins1* in β cells cultured in 33.3 mmol/L glucose, but also was related to upregulated dedifferentiated cells markers NGN3 and OCT4 (Fig. 1e-l), indicating a significant correlation between impaired GSIS and compromised β-cell identity. Subsequently, we found that a high glucose concentration triggered RAS signaling, which could be inhibited by Irbesartan, an AT1R blocker. Insulin secretion from β-cell stimulated with 25 mmol/L of glucose in the IRB-treated group was slightly improved compared with that in β cells cultured in the high glucose environment (22.2 mmol/L or 33.3 mmol/L, Fig. 1a-b). In addition, IRB enhanced the stimulatory index in INS1 cells under 22.2 mmol/L glucose conditions (Fig. 1c). The inhibitor improved GSIS and markedly reduced the mRNA expression of *Ngn3*, especially in 22.2 mmol/L glucose. We observed an increasing trend of the stimulatory index in min6 cells, although there was no significant change, and the basal, as well as stimulated, insulin secretion was significantly promoted by Irbesartan in the high glucose environment (22.2 mmol/L or 33.3 mmol/L).

### The deleterious effect of Angll is dependent on NF-κb signaling in β cells

**Fig. 2:**
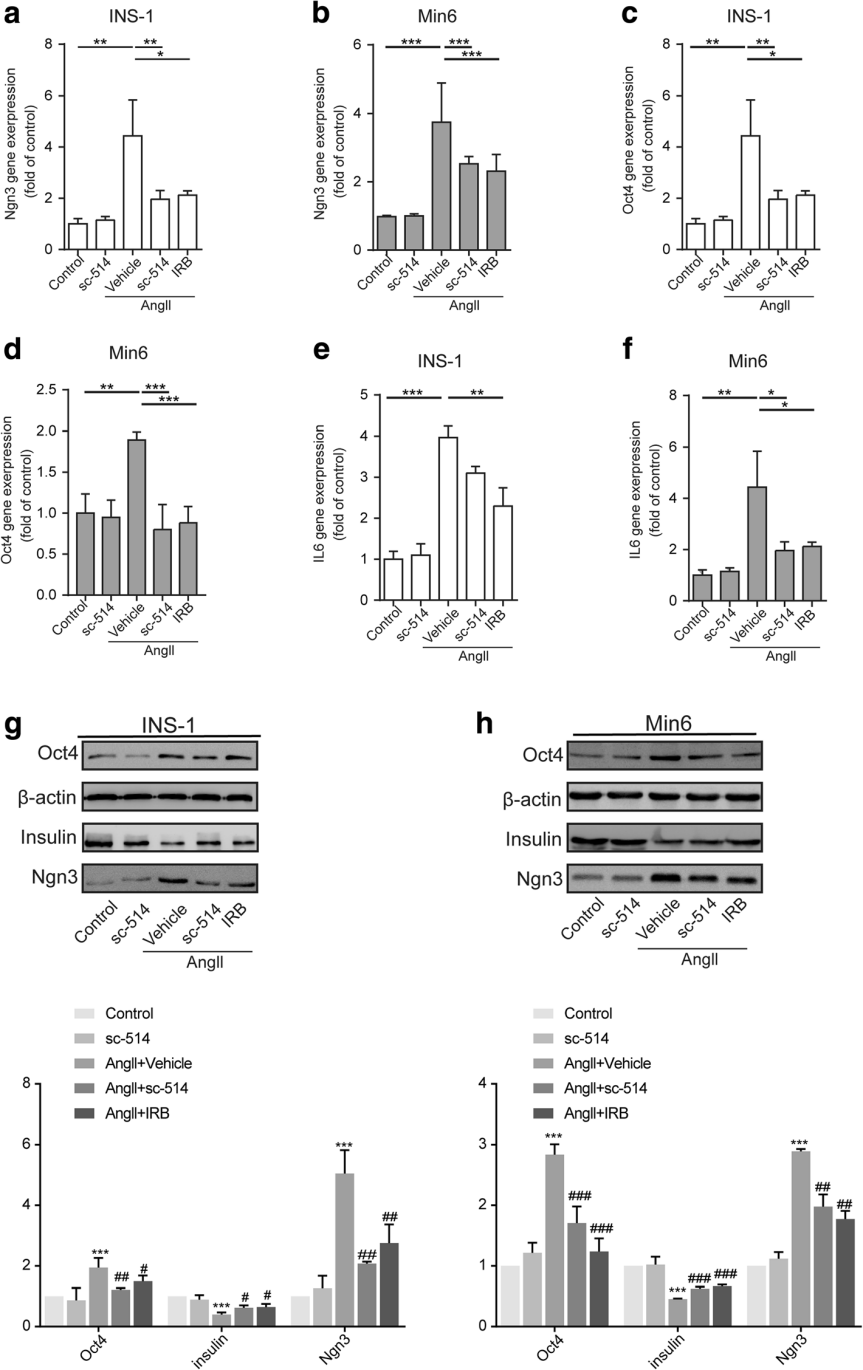
The deleterious effect of Angll is dependent on NF-κb signaling in β cells. Pancreatic β cell lines were cultured with or without Angll (1 μmol/L) in the presence or absence of sc-514, an IkB-kinase-2 inhibitor (20 μmol/L), or Irbesartan (IRB) (10 μmol/L) for 48 h. qRT-PCR analyses for (**a-d**) progenitor likes cell markers (*Ngn3*, *Oct4*) and (**e, f**) proinflammatory cytokine *Il6*. Data are presented as the mean ± SEM of three independent experiments (*n* = 6), *p < 0.05, **p < 0.01, ***p < 0.001, one-way ANOVA. **g, h** Western blotting for NGN3, OCT4, and insulin in Min6 cells and INS-1 cells. β-actin was used as a loading control. Densitometric analyses of the western blotting results are presented below. Data are presented as the mean ± SEM of three independent experiments(*n* = 6). **p < 0.01, ***p < 0.001 vs Control group, ^**#**^*p* < 0.05, ^**##**^*p* < 0.01, ^**###**^*p* < 0.001 vs Vehicle group, one-way ANOVA

To identify the role of RAS activation in β-cell dedifferentiation, we cultured INS-1 and Min6 cells with or without AngII (1 μmol/L) in the presence with Irbesartan or with sc-514. As observed in β cells, Angll obviously increased the gene expression of *Ngn3*, *Oct4*, and *IL6* compared with that in the control group (Fig. 2a-f). Meanwhile, the dedifferentiation and proinflammatory effects of Angll on β cells were significantly attenuated by Irbesartan. Similarly, sc-514, an IkB-kinase-2 inhibitor, markedly decreased the Angll-induced dedifferentiation level. Furthermore, we investigated the protein expression levels of dedifferentiation markers NGN3, OCT4, and insulin in the indicated groups, to examine the differentiation stage of β cells (Fig. 2g, h). As expected, AngII increased the levels of NGN3 and OCT4, while Irbesartan and sc-514 both efficiently blocked NGN3 and OCT4, especially in Min6 cells. Meanwhile, Irbesartan and sc-514 restored the expression of Insulin. Therefore, inhibiting IkB-kinase reversed the dedifferentiation effect of Angll, which provided evidence that compromised β-cells identity is associated with NF-κb signaling.

### Angll induces the activation of NF-κb, leading to dedifferentiation and dysfunction in β cells

**Fig. 3:**
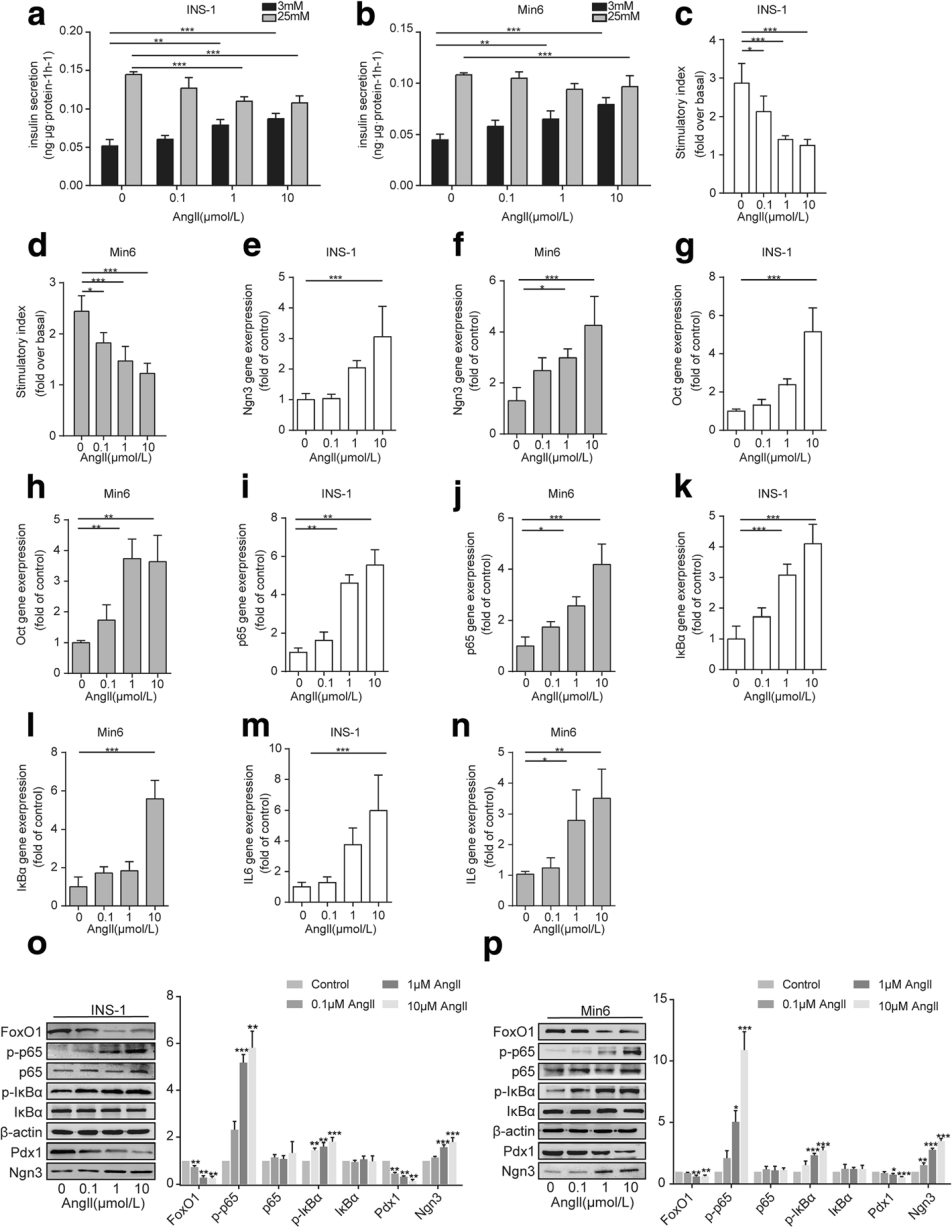
Angll induces the activation of NF-κb, leading to dedifferentiation and dysfunction in β cells. Pancreatic β cell lines were cultured with increasing doses of Angll for 48 h. Performing (**a, b**) a GSIS assay to determine (**c, d**) the stimulatory index in Min6 cells and INS-1 cell. qRT-PCR analyses for (**e-h**) progenitor like cells markers (*Ngn3*, *Oct4*), (**i-l**) NF-κb signaling (*lκBα*, *p65*) and (**m, n**) proinflammatory cytokines *Il6* in Min6 cells and INS-1 cell. Data are presented as the mean ± SEM of three independent experiments (*n* = 6), *p < 0.05, **p < 0.01, ***p < 0.001, one-way ANOVA. **o, p** Western blotting for P65, IκBα, PDX1, NGN3, FOXO1, and the phosphorylated forms of P65 (p-P65) and IκBα (p-IκBα) in Min6 cells and INS-1 cells. β-actin was used as a loading control. Densitometric analyses of the western blotting results are presented. Data are presented as the mean ± SEM of three independent experiments. (*n* = 6) *p < 0.05, **p < 0.01, ***p < 0.001 vs control group, one-way ANOVA

We further investigated the relationship between NF-κb signaling and Angll-dependent β-cell dedifferentiation. Pancreatic β cells treated with increasing doses of Angll showed a remarkable increase in basal insulin secretion and a dose-dependent decline in the stimulatory index (Fig. 3a-d). We also found that Angll activated NF-κb signaling via the phosphorylation of p65 and IκBα, in a dose-dependent manner. Meanwhile, β cells that had lost their identity displayed decreased expression of PDX1 and FOXO1, and increased NGN3 expression (Fig. 3o, p). Moreover, consistent with the change in protein expression, we found that the mRNA expression levels of *p65*, *IκBα*, *Ngn3*, and *Oct4* were positively correlated with the Angll dose in β cells (Fig. 3e-l). Interestingly, we found that IL6 was significantly increased when the cells were incubated with 10 μm/L Angll, indicating the proinflammatory effect of Angll (Fig. 3m, n).

### Angll treatment of diabetic mice potentiated impaired glucose tolerance and compromised β-cell identity

**Fig. 4:**
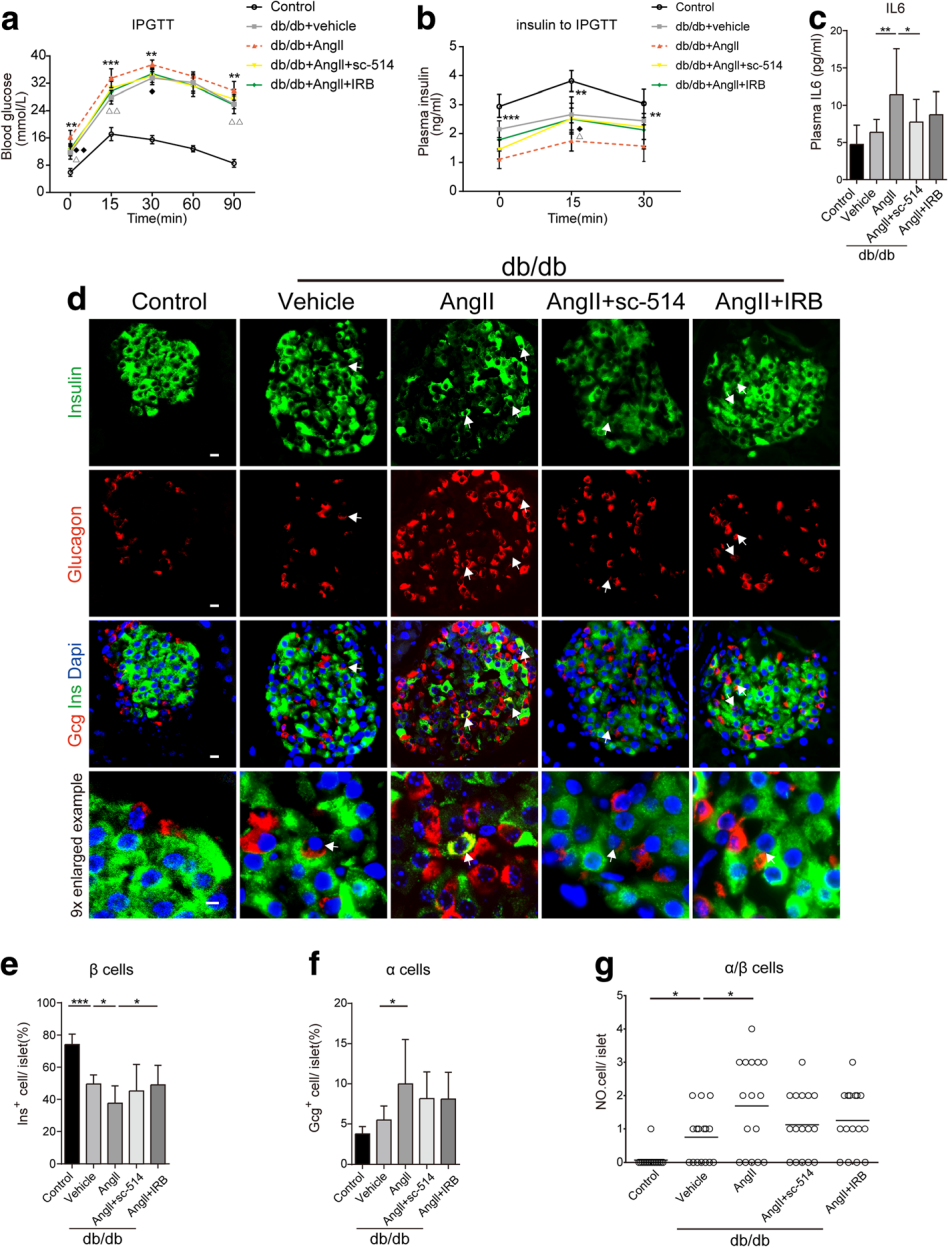
(color; online only) Angll treatment of diabetic mice potentiates impaired glucose tolerance and compromised β-cell identity. db/db mice were injected with Angll (60 μg/kg) subcutaneously twice a day for 4 weeks. Where indicated, either sc-514 (30 mg/kg) or Irbesartan (IRB) (50 mg/kg) was also administered. **a** Glucose levels in homeostasis and intraperitoneal glucose tolerance tests (IPGTTs) for the indicated groups (*n* ≥ 6 for each group). **b** Levels of circulating insulin during IPGTT (*n* = 6 for each group). Data (**a, b**) are presented as the mean ± SEM, *p < 0.05, **p < 0.01, ***p < 0.001 (Angll treated vs. db/db control); ^♦^*p* < 0.05, ^♦♦^*p* < 0.01 (Angll treated vs. sc-514 treated); △*p* < 0.05, △△*p* < 0.01, (Angll treated vs. IRB treated), as assessed using repeated measures ANOVA (**c**) Plasma IL-6 measurements (*n* ≥ 6 for each group). Mean ± SEM,*p < 0.05, **p < 0.01, ***p < 0.001, one-way ANOVA (**d**) Paraffin embedded sections from the indicated groups were immunolabeled for insulin (green), glucagon (red), and DAPI (blue). Scale bar (10 μm). Arrow in the 9× enlarged example image indicates a typical insulin^+^glucagon^+^ (Ins + Gcg+) cell. Scale bar (5 μm) in the 9× enlarged example image. **e, f** Quantification of labeled insulin or glucagon positive cells per islet. **g** Numbers (NO.) of insulin^+^glucagon^+^ cells. For Immunofluorescence analysis (e-g): *n* ≥ 4 mice per group, *n* ≥ 20 islets per marker. Mean ± SEM *p < 0.05, **p < 0.01, ***p < 0.001, one-way ANOVA

Next, type 2 diabetic mice models (db/db mice) and wild-type controls (db/m mice) were used to identify the role of Angll in the deterioration of β-cell function in vivo. The mice were randomly divided into five groups. The diabetic mice were injected with Angll (60 μg/kg) or vehicle subcutaneously twice a day for 4 weeks and orally administered with sc-514 (30 mg/kg) or Irbesartan (50 mg/kg) at the same time. After 4 weeks, an intraperitoneal glucose tolerance test (IPGTT) showed elevated blood glucose and reduced plasma insulin concentration in db/db mice treated with Ang II alone compared with that in control db/db mice, suggesting impaired glucose tolerance induced by Ang II (Fig. 4a-b). The body weight of db/db mice at 9 weeks of age was significantly higher than that of wild-type littermates. Meanwhile, we did not observe significant difference between the db/db control group and db/db treatment groups (Additional file 2: Figure S1). In addition, we observed increasing release of cytokine IL6 in the AngII-treated mice, suggesting an AngII-induced proinflammatory effect that can be attenuated by an IkB-kinase-2 inhibitor (Fig. 4c). Irbesartan or sc-514 slightly restored the impaired glucose tolerance caused by AngII and thus decreased blood sugar levels. Moreover, progressive loss of islet β cells during Angll treatment resulted in a significantly low β-cell ratio, which decreased by 12% per islet, as well as an increased glucagon-positive islet ratio. Conversely, the loss of insulin positive cells could be reversed by Irbesartan. (Fig. 4d-f). Intriguingly, mice treated with AngII alone had comparably increased numbers of cells co-expressing both glucagon and insulin to that in the control db/db mice (Fig. 4g), suggesting that Angll induced β cells to convert into α cells.

### Sc-514 reversed the β-cell dedifferentiated state and dysfunction in db/db mice

**Fig. 5:**
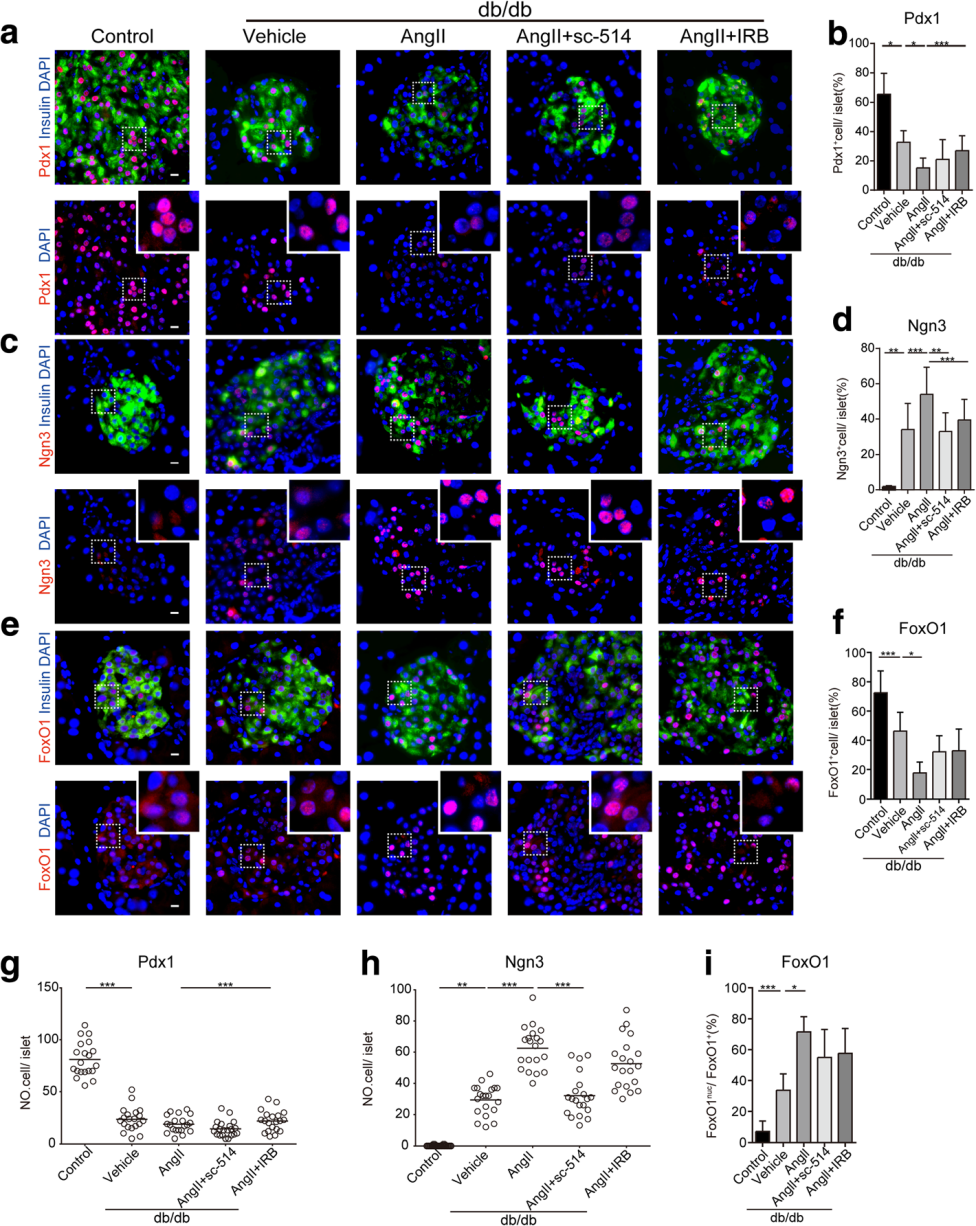
(color; online only) sc-514 reversed β-cell dedifferentiation and dysfunction in db/db mice. **a**, **c**, **e** paraffin-embedded sections from the indicated groups were immunolabeled for insulin (green), PDX1 (red), insulin (green), NGN3 (red), insulin (green), FOXO1 (red), and DAPI (blue) (**b, d, f**) Quantification of labeled PDX1 or NGN3 or FOXO1 positive cells per islet. The squares (dashed white lines) show regions 9× enlarged and depicted as inserts at the top right of the corresponding images. **g, h** Numbers (NO.) of PDX1 or NGN3 positive cells. Scale bar (10 μm). (**i**) Quantification of FOXO1 translocated to the nuclei of FOXO1-positive cells. For the immunofluorescence analysis: *n* ≥ 4 mice per group, n ≥ 20 islets per marker. Mean ± SEM *p < 0.05, **p < 0.01, ***p < 0.001, one-way ANOVA

We further assessed whether Angll-induced β-cell failure is associated with the activation of NF-κb signaling. The proportion of PDX1 (Fig. 5a, b) positive cells markedly decreased by 17% and the percentage of Ngn3 (Fig. 5c) positive cells increased to 53% in mice with chronic activation of RAS compared with that in the control db/db mice Meanwhile, the number of PDX1 or NGN3 positive cells was consistent with their ratio change (Fig. 5g, h). In addition, FOXO1 was mainly localized in the cytoplasm of β cells in normal control mice, whereas it was translocated into the nucleus in db/db mice (Fig. 5e). In db/db mice treated with Angll, we found a loss of expression of FOXO1 (Fig. 5f) and increased nuclear-localized FOXO1 protein compared with control db/db mice (Fig. 5i). Furthermore, blocking RAS with Irbesartan partially reversed β-cell failure and marginally increased the islet PDX1-positive ratio by 12% (Fig. 5b). Meanwhile, in the sc-514 treated group, there was an upward trend in the islet PDX1-positive ratio compared with that in the control db/db group, although we observed no detectable differences in the ratio between these two groups. In addition, we observed that sc-514 decreased the NGN3 positive ratio by 20% (Fig. 5d), which was 7% higher than that in mice treated with Irbesartan. The results indicated that Irbesartan rescues the loss of β cells and restores β-cell identity; largely depend on enhanced key β-cell transcription factors. Moreover, sc-514 improves β-cell function mainly by suppressing the expression of NGN3, and thus reverses β-cell dedifferentiation.

## Discussion

**Fig. 6:**
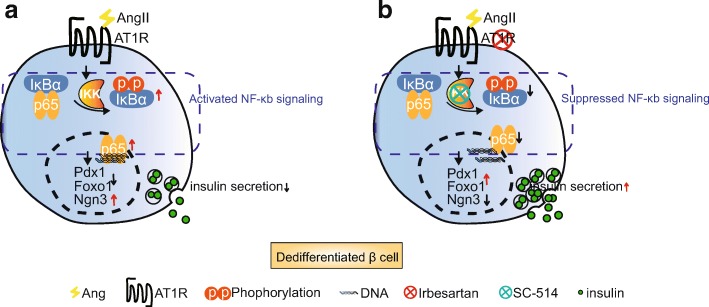
(color; online only) **(a)** Metabolic stress (i.e., oxidative stress, proinflammatory cytokines) is a major contributor to β-cell dedifferentiation, and activation of RAS is involved in induction of metabolic stress. Angll contributes to β cell dedifferentiation via activating AT1R and the contribution is supported by the activation of NF-κb signaling. This process results in compromised β cell identity and β cell dedifferentiation (PDX1↓FOXO1↓NGN3↑), thus decreasing insulin secretion. **b** When NF-κb signaling is suppressed by sc-514 (IKK inhibitor), the dedifferentiation effect of Angll on β cells is alleviated (PDX1↑FOXO1↑NGN3↓), resulting in increased insulin secretion. A RAS inhibitor showed a similar effect on β cells

Growing evidence suggests that activation of RAS increases oxidative stress and impairs insulin secretion in islets (Chan et al., 2017; Sauter et al., 2015). Accordingly, blocking RAS helps to protect β cells from glucotoxicity and leads to functional improvement (Wang et al., 2011). Studies have shown that metabolic stress is a potential underlying cause of β-cell dedifferentiation (Guo et al., 2013a; Talchai et al., 2012a), which is an important contributory factor to β-cell failure (Cinti et al., 2016; White et al., 2013). However, the relationship between RAS and the dedifferentiated status of β cells remains elusive. Our study, for the first time, found that Angll contributes to β-cell dedifferentiation via AT1R activation in diabetic mice. Moreover, this contribution is strengthened by NF-κb signaling (Fig. 6).

High glucose levels are known to be closely associated with β-cell dysfunction in type 2 diabetes, and abnormal glucose-stimulated insulin secretion (GSIS) can explain the mechanism. In addition, High glucose levels can cause glucotoxicity, which reduces β-cell-identity markers, including MAFA, PDX1, and lNS1 (Jonas et al., 1999; Kondo et al., 2009), and triggers RAS. In our study, we confirmed that lrbesartan, a RAS inhibitor, markedly reversed impaired GSIS, and downregulated dedifferentiated cells markers in a high glucose environment. Subsequently, we identified the role of RAS in β-cell dedifferentiation.

Recent studies have reported that pancreatic β cells become dedifferentiated and convert to other endocrine cells under certain circumstances (Chakravarthy et al., 2017; Thorel et al., 2010) Consistently, we demonstrated that Angll efficiently induces the conversion of β cells into glucagon-producing α-like cells in db/db mice, which might explain the phenomenon that patients with type 2 diabetes are more likely to have elevated plasma glucagon levels and a glucagon-to-insulin ratio (Dunning and Gerich 2007). Although we did not observe a significant effect of treatment (sc-514 or IRB) on the number of α/β cells, there was a decreasing trend of α/β cells compared with that in the Angll-treated db/db group. In addition, we found a reversed insulin-positive ratio and a decreasing trend in glucagon-positive cells in the Irbesartan-treated group, which both indicate the reversible identity of β cells, as well as the potential conversion of other types of pancreatic cell into β-cells. However, the specific types of pancreatic cell involved need to be clarified. On the basis of previous studies, we investigated Angll, a RAS signaling component that functions as a promoter of inflammation, fibrosis, and apoptosis via the angiotensin II type 1 receptor (AT1R) in many tissues (Miyazaki and Takai 2006; Paul et al., 2006). Consistently, we observed increased serum IL6 and loss of insulin positive cells in AngII-treated diabetic mice, which suggested the proinflammatory effect of Angll. In addition, several studies have demonstrated the possible vasoconstrictive effect of Angll on glucose and insulin kinetics. Recently, Sauter et al. (2015) found that Angll induced impaired glucose tolerance and insulin secretion, independent of its vasoconstrictive effects. The authors ruled out the vasoconstrictive effect of Angll by treatment with hydralazine, a direct-acting vasodilator. Furthermore, the study suggested that Angll leads to islet dysfunction via induction of inflammation; however, they did not conduct further experiments to determine the status of β cells. Therefore, the study shed a light on the vasoconstrictive effect of Angll, which might not be a confounding factor in impaired glucose tolerance, and the underlying mechanism of AngII dysfunction was not fully clarified. The present study might be considered as a hypothesis to observe the effect of direct activation of RAS induced by Angll on the state of β cells. We detected the differentiation effect of Angll in β cell lines, manifested by downregulation of β-cell identity markers and a decrease in glucose-stimulated insulin secretion. We then examined the dedifferentiation effect of directly triggering RAS on β cells in diabetic mice. The results showed a loss of positive areas for PDX1 and FOXO1 and high expression of NGN3, indicating the development of dedifferentiation in β cells, although we failed to identify the possible vasoconstrictive effect of Angll on glucose and insulin in db/db mice. The loss of mature status of β cells is detrimental to their identity, ultimately leading to β-cell dysfunction.

The expression pattern of FOXO1 during pancreatic organogenesis is identical to that of PDX1 (Kitamura et al., 2009). FOXO1 increases the expression of transcription factor HES-1, which is a repressor of NGN3. Consequently, in *FoxO1* knockout mice, *Ngn3* expression is upregulated in gut endocrine cells (Talchai et al., 2012a, b), suggesting that FOXO1 essentially prevents β-cell differentiation. Meanwhile, we found that FOXO1 translocates from the cytoplasm to the nucleus in response to Angll, which was consistent with previous reports that FOXO1 is a malfunctional protein involved in insulin signaling and translocation in β cells when faced with oxidative stress (Kitamura 2013; Kitamura et al., 2005). lrbesartan slightly promoted these effects and rescued the loss of insulin positive cells by increasing the numbers of insulin positive cells in Angll-infused db/db mice. Importantly, these data suggested that Angll-induced RAS activation is a major contributor to dedifferentiation, which can be reversed by RAS inhibitors, resulting in restoration of β-cell function.

Studies have reported that activation of NF-κb occurs through AT1R (Luo et al., 2015; Thomas et al., 2014). In a diabetic mice model treated with Angll, Sauter et al. (2015) found that NF-κb signaling meditated inflammation and participated in Angll-induced deterioration of glucose metabolism, suggesting an interaction between RAS and NF-κb in hypertension and diabetes. The present study showed that blockade of NF-κb using sc-514 reversed dedifferentiation by decreasing the NGN3-positive area by 20%. Consequently, we proved that sc-514 promotes glucose metabolism by reversing the differentiated state of β cells, without affecting the number of β cells. In the present study, we observed a strong inhibitory effect of Irbesartan and sc-514 on the suppression of dedifferentiation of β cells in Angll-treated db/db mice. Additionally, NGN3 levels increased significantly in the db/db control group compared with those in the control group. This corresponded with the compromised β-cell identity in patients with type 2 diabetes. It would be interesting to determine whether IRB or NF-κb inhibition also result in an improvement in the identity of β cells in the db/db control group. Considering the notable effect we observed in vivo and the potential clinical significance, this is a direction worthy of further study.

Recently, Cinti et al. (2016) hypothesized that β-cell dedifferentiation could be a mechanism to protect β cells from undergoing apoptosis, enabling them to redifferentiate under more favorable circumstances. In support of this, we showed that β cells lose their differentiated characteristics under metabolic stress, eventually leading to compromised function. However, the progressive impairment of β cells is reversible. Taken together, this evidence suggests that dedifferentiation-driven β-cell failure can be reversed under certain circumstances. Meanwhile, amelioration of insulin secretion by residual cells, such as differentiated cells, is likely to be a rapid way to restore β-cell function.

## Conclusions

In summary, we propose a pathway lead from chronic RAS accumulation to NF-κb signaling that eventually causes β-cell dedifferentiation. Our findings prove that RAS induces pancreatic β-cell dedifferentiation and provide pharmacological strategies to reverse dedifferentiation by suppressing NF-κb signaling.

## Additional files

Additional file 1: **Table S1.** Body weight development in each group. Data are presented as the mean ± SEM (*n* = 8 each) ***p < 0.001 vs control group, one-way ANOVA. DM, db/db mice; Age (weeks). (PDF 183 kb)

Additional file 2: **Figure S1.** Related to Fig. 1. Pancreatic β cell lines were cultured in the presence or absence of irbesartan (IRB, 10 μmol/L) for 24 h. Performing a GSIS assay to determine the stimulatory index in Min6 cells and INS-1 cells. qRT-PCR analyses for markers of β cell identity genes, and progenitor like cells markers in β cells. Data are presented as the mean ± SEM of three independent experiments (*n* = 6). (TIF 1669 kb)

## Abbreviations

ACEi: Angll-converting enzyme inhibitors
AngII: Angiotensin II
ARBs: Angiotensin II type 1 receptor blockers
AT1R: Angiotensin II type 1 receptor
GSIS: Glucose-stimulated insulin secretion
IKK: IкB kinase
IL-1b: Interleukin-1b
IL6: Interleukin-6
IRB: Irbesartan
KRBH: Krebs–Ringer bicarbonate HEPES
RAS: Renin–angiotensin system
