# Supplementary material for: Reversal of angiotensin ll-induced β-cell dedifferentiation via inhibition of NF-κb signaling
Source: Mol Med. 2018 Aug 14;24:43. doi: 10.1186/s10020-018-0044-3 (PMC6092859; doi:10.1186/s10020-018-0044-3)
Supplement: Supplementary file 1 — Table S1. Body weight development in each group. Data are presented as the mean ± SEM (n = 8 each) ***p < 0.001 vs control group, one-way ANOVA. DM, db/db mice; Age (weeks). (PDF 183 kb) [file 10020_2018_44_MOESM1_ESM.pdf]

TableS1 Body weight development in each group.

	Control	DM	DM+AngII	DM+AngII+sc-514	DM+AngII+IRB
Age (w)			Weight (g)		
8	22.8 ± 1.06	38.5 ± 2.48***	37.1 ± 1.04***	37.8 ± 2.10***	38.2 ± 2.22***
9	23.3 ± 1.02	40.1 ± 2.57***	38.6 ± 0.91***	39.2 ± 2.14***	39.6 ± 2.32***
10	24.0 ± 0.92	41.7 ± 2.32***	40.7 ± 1.34***	40.9 ± 1.90***	41.2 ± 2.23***
11	24.7 ± 1.00	43.4 ± 2.10***	42.6 ± 0.92***	42.8 ± 1.58***	42.6 ± 2.06***
12	25.4 ± 0.99	44.2 ± 2.13***	43.4 ± 0.88***	43.6 ± 1.69***	43.7 ± 2.27***

Data are presented as the mean ± SEM (n=8 each) ***p < 0.001 vs control group, one-way ANOVA. DM，db/db mice.
