# Supplementary figures and images for: Reversal of angiotensin ll-induced β-cell dedifferentiation via inhibition of NF-κb signaling
Source: Mol Med. 2018 Aug 14;24:43. doi: 10.1186/s10020-018-0044-3 (PMC6092859; doi:10.1186/s10020-018-0044-3)

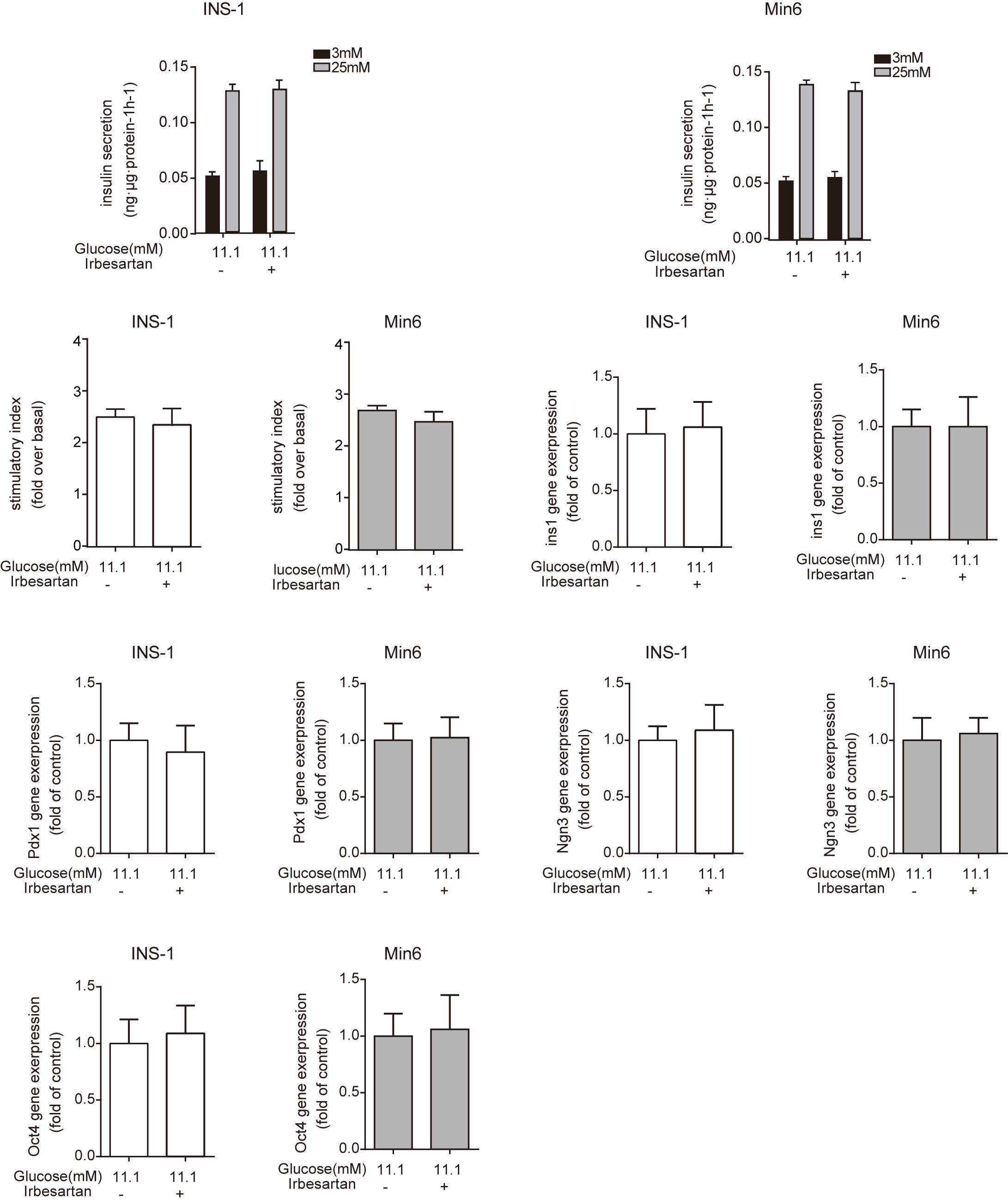

Supplement: Supplementary file 2 — Figure S1. Related to Fig. 1. Pancreatic β cell lines were cultured in the presence or absence of irbesartan (IRB, 10 μmol/L) for 24 h. Performing a GSIS assay to determine the stimulatory index in Min6 cells and INS-1 cells. qRT-PCR analyses for markers of β cell identity genes, and progenitor like cells markers in β cells. Data are presented as the mean ± SEM of three independent experiments (n = 6). (TIF 1669 kb) [file 10020_2018_44_MOESM2_ESM.tif]
